# Oxygen Sensing, Cardiac Ischemia, HIF-1α and Some Emerging Concepts

**DOI:** 10.2174/157340310793566136

**Published:** 2010-11

**Authors:** Shyamal K Goswami, Dipak K Das

**Affiliations:** Cardiovascular Research Center, University of Connecticut School of Medicine, Farmington, Connecticut, CT 06030- 1110, USA

**Keywords:** Hypoxia, oxygen sensing, HIF-1, prolyl hydroxylase, cardiac ischemia, preconditioning.

## Abstract

Oxygen plays a critical role in the perpetuation and propagation of almost all forms of life. The primary site of cellular oxygen consumption is the mitochondrial electron transport chain and in addition, oxygen is also used as a substrate for various enzymes involved in cellular homeostasis. Although our knowledge of the biochemistry and physiology of oxygen transport is century old, recent development of sophisticated tools of biophysical chemistry revealed that tissue oxygenation and oxygen sensing is a highly evolved process, especially in mammals. Perturbation of normal oxygen supply is associated with diseases like tumorigenesis, myocardial infarction and stroke. Available information suggests that when tissue oxygen supply is limited, mitochondria emanate signals involving reactive oxygen species generation which in turn stabilizes oxygen sensing transcription factor HIF-1. Upon stabilization, HIF-1 elicits necessary genetic response to cope with the diminished oxygen level. In view of such critical role of HIF-1 in cellular oxygen sensing, recently there has been a heightened interest in understanding the biology of HIF-1 in the context of cardiovascular system. The following review describes some of the recent advances in this regard.

## INTRODUCTION

Molecular oxygen, perhaps is only second to water for its critical role in the sustenance and propagation of life process. Nevertheless, although the biochemical mechanisms of oxygen transport (by hemoglobin) and its consumption in the mitochondria (by oxidative phosphorylation) was established almost half a century ago, the comprehensive understanding of its role in aerobic life is still emerging [1, 2]. Data accumulated over the years suggest that apart from its key role in ATP generation, oxygen is also involved in cellular processes such as biosynthesis of sterol and prostaglandin, detoxification by cytochrome p-450 etc [3, 4]. In addition, cellular oxygen is also attributed to the intracellular generation of a plethora of highly reactive molecules collectively known as reactive oxygen/nitrogen species (ROS/RNS) causing oxidative modifications of various biomolecules with diverse consequences [5-7]. Taken together, it is expected that oxygen homeostasis, as maintained by cellular oxygen uptake and consumption, have profound effects on cellular wellbeing [1]. Accordingly, inadequacy of oxygen supply or hypoxia, either to the whole body or to certain tissues has severe pathological consequences such as cardiovascular diseases, tumorigenesis and stroke [8]. 

Our interest in the pathobiology of hypoxia dates back to nineteen thirties (with first Pubmed entry in 1939) and since it has been one of the most extensively investigated subject in modern biology (with 79802 Pubmed entries till March, 2008). However, the molecular insight into the hypoxic response was first gained only in early nineties in the context of tumorigenesis, while that by the cardiovascular system| came thereafter. The present review is thus aimed towards summarizing some of those recent observations and putting them in the larger context of the physiology of oxygen sensing by the myocardium. 

### A Tissue Oxygen Level and Oxygen Sensing

Mammalian tissues are characterized by aerobic metabolism and require uninterrupted oxygen supply. Accordingly, availability (or otherwise) of oxygen in tissues play a critical role in the pathophysiology of the organism [9]. However, precise measurement of oxygen levels in various organs is a challenging task and our understanding of tissue oxygen levels is still inadequate [10]. Available data also suggests that partial pressure of oxygen in various tissues might vary significantly. According to one study in rat, oxygen tension is highest in the bladder (~ 60 mmHg), followed by muscle (~40 mmHg), liver (~20 mmHg), and renal cortex (~ 15 mmHg) [9]. Since the availability and consumption of oxygen in various tissues may vary under different pathophysiological conditions, the respiratory process involving the heart, lung and the brain finely tunes the supply and demand of tissue oxygen while failure in such regulation leads to hypoxia (or hyperoxia). Hypoxic condition may arise under a number of conditions such as low partial pressure of oxygen in arterial blood (as in case of pulmonary diseases or high altitudes); reduced ability of the blood to carry oxygen (as in case of anemia); reduced tissue perfusion of oxygen (as in case of ischemia) and altered geometry of the tissue and the microvessels (as in case of tumors) [11]. Taken together, it is thus imperative that mammalian cells have acquired an elaborate mechanism to sense tissue oxygen supply and take remedial measures under hypoxia or heperoxia. 

At the organismal level, oxygen sensing is done by the carotid body, located along with the carotid artery that supplies freshly oxygenated blood to the brain. Glomus cells of the carotid body senses diminished oxygen supply and release dopamine. Dopamine then activates the sensory neurons, initiating a series of responses by the respiratory and cardiovascular system ensuring adequate oxygenation of all the organs [12]. Mechanisms of oxygen sensing by the individual tissues and cells are more diverse and are often debated [13]. Diverse types of proteins/enzymes like potassium channels, mitochondrial complex III and IV, NADPH oxidases, prolyl hydroxylases and heme oxygenases have been described as oxygen sensors in various cellular contexts [14]. Oxygen sensitive potassium channels are found in carotid body, lung, adrenal medulla and smooth muscle cells [15-17]. However, in spite of convincing evidences about their oxygen sensing functions, it has also been argued that potassium channels are not the “oxygen sensors” as such, but are coupled to some other sensing molecules located in the plasma membrane [18-20]. In this context, intracellular reactive oxygen species generation that depends upon the state of oxygenation, have often been considered as the oxygen sensors for potassium channels and other oxygen sensitive pathways [20, 21]. A number of hypoxia sensitive channels including I_Ks_ have been identified in cardiac myocytes [21]. Oxygen level and the duration of hypoxia play critical roles in determining responses elicited by the cardiac channels. However, many cardiac ion channels do not respond to hypoxia [21]. 

### B Oxygen Sensing in Heart and Flow Regulation

Mammalian heart is an obligatory aerobic organ consuming large amount of oxygen for energy generation and contractile functions [22]. The rate of oxygen utilization by the myocardium further increases upon vigorous exercise. Heart also requires oxygen for synthesizing certain regulatory molecules like nitric oxide and other reactive species playing critical roles in cardiovascular biology. Cardiac function is thus severely impaired upon limiting (as well as surplus) oxygen supply and cardiologists have been looking for a comprehensive understanding of the delivery of oxygen to the myocardium [23]. Coronary vasculature plays an important role in myocardial oxygen sensing. Decrease in arterial oxygen content (or increase in myocardial work load) is immediately sensed by the smooth muscle cells in the vessel wall resulting in an instantaneous increase in coronary flow, ensuring adequate oxygen supply [24]. Available evidences suggest that even slight changes in coronary oxygen level leads to altered energy metabolism, which in turn modulate coronary tone/flow in a complex manner [25]. Furthermore, although reduction in oxygen concentration results in a decrease in the respiratory rate and [ATP]/[ADP] ratio; even at low O_2 _tensions, the mitochondrial respiratory chain remains at near equilibrium with the ATP synthesizing reactions [26, 27]. Thus, metabolic pathways play a role in tissue oxygen sensing and limited oxygen supply affects cellular metabolism much before it affects respiration. A number of metabolites have thus been investigated for their crucial roles in vascular oxygen sensing and flow regulation. When rat heart coronary vessel preparations are exposed to hypoxia, vessels surrounded by myocardial tissues are dilated to a greater extent than those which are stripped [28]. Available evidences suggest that under hypoxic condition, endothelial cells produce prostaglandin followed by vasorelaxation [29, 30]. Hypercapnic acidosis, a condition arising due to the accumulation of carbon dioxide-decrease in pH due to reduced oxygen supply, has also been attributed to the regulation of coronary flow while nitric oxide, adenosine and K_ATP_ channels have been coined as possible mediators [31, 32]. However, whether or not those metabolites play a universal role in arterial oxygen sensing under different physiological/experimental conditions is a subject of intense debate [24]. 

### C Myocardial Response to Hypoxia

Myocardium might undergoes hypoxia (or anoxia) under a number of pathophysiological conditions like coronary artery occlusion, anemia and at high altitude [22, 23]. Also, under hypoxic conditions, the availability of oxygen in various parts of the myocardium might remain uneven [33]. Due to the criticality of oxygen supply, cardiac cells afflicted by mild hypoxia often readjust their oxygen sensing “set point” while restoration of normal oxygen level results in a condition termed as “perceived hyperoxia” [23]. Cardiac response to diminished oxygen supply thus depends upon its extent, duration, as well as other associated factors (such as ischemia); while measurable reduction in the cellular oxygen utilization (by cytochrome c oxidase) occurs only upon substantial depletion of oxygen supply [34-38]. During the past decades, investigators have used different experimental settings and diverse biochemical-molecular markers to understand the effects of hypoxia on the cardiovascular system and thus had accumulated wealth of information in this regard. Studies with cultured myocytes have shown that hypoxia induces endoplasmic reticulum stress wherein AMPKinase play a protective role [39, 40]. Also in hypoxic myocytes, induction of adrenomedullin (pro-survival peptide hormone), Akt (pro-survival kinase), BNip3 (pro-death BH3 domain containing protein) and Gene 33/RALT (pro-death adapter protein) have been reported [41-44]. In contrast to cultured myocytes, studies with whole heart either ex vivo or *in vivo* had shown divergent and often conflicting results. Mice undergoing acclimatization to long term normobaric hypoxia (10% O_2_) expressed heme oxygenase 1/2 in the heart, while those undergoing short term acute hypobaric hypoxia (426 mm Hg) showed altered expression of both pro- and anti oxidant genes [45, 46]. According to one study, adult male rats exposed to 10% inspired O_2_ (daily 6 hours) showed cardioprotection (*via* augmentation of RyR and NCX) when isolated perfused hearts were subjected to I/R [47]. On the contrary, intermittent hypoxia (5% inspired oxygen for 40 sec followed by infusion of compressed air for 20 sec) for four hours increased sensitivity to reperfusion injury that was preventable by recombinant erythropoietin [48]. In a more elaborate study, where rats were subjected to brief ischemia by coronary ligation showed differential regulation of battery of genes viz., BNP, HIF-1, IL-6, iNOS, IGF, Prepro-endothelin-1, sarcoplasmic reticulum Ca2+ ATPase, phospholamban and Na+-Ca2+ exchanger etc., in the left and right ventricle [49]. Taken together, depending upon of mode and duration of hypoxia, myocardial response could be complex and involve integration of multiple signaling pathways with various consequences [50, 51]. 

### D Mitochondria and Cellular Oxygen Sensing

Mitochondria is the primary site for cellular oxygen consumption wherein it binds to cytochrome oxidase and receives electrons from reduced cytochrome c. Constituents of electron transport chain in general and cytochrome oxidase in particular have thus been critically examined for their potential role(s) in oxygen sensing, albeit with conflicting inferences [52]. Early studies had shown that the pO_2_ required for half-maximal reduction of cytochrome c can be as low as 0.2-0.02 Torr, thereby making it more suited for sensing anoxia rather than hypoxia [53,54]. In agreement, it has also been observed that inhibition of oxidative phosphorylation (and thus of cytochrome c) by cyanide and antimycin does not affect the hypoxic induction of erythropoietin mRNA [55]. On the contrary, it has also been demonstrated that under prolonged hypoxia, the catalytic activity of cytochrome c oxidase is inhibited by an allosteric mechanism, thereby supporting the notion that it is an oxygen sensor [56, 57]. Thus, in spite of an early consensus on the criticality of mitochondria in oxygen/hypoxic sensing, the precise nature of the sensor (and its mechanism of action) remained elusive for quite sometimes. The criticality of mitochondria in eliciting hypoxic response was further revealed upon demonstration that it requires intact mitochondrial genome and involves reactive oxygen species generation [58-60]. Although the essentiality of intact mitochondrial electron transport chain in hypoxic sensing has since been disputed [61, 62], possible explanation against such discrepant observations has also been offered [63]. Taken together, the mechanism(s) of oxygen sensing by mitochondria is yet to be fully understood. However, as discussed below, studies over the past decade have convincingly established the existence of an axis of mitochondrial reactive oxygen species generation and hypoxic response. 

### E Mitochondrial Reactive Oxygen Species Generation and Cardiovascular Diseases 

Mitochondrial electron transport chain is a complex assembly redox-proteins engaged in transfer of electrons from NADH/FADH_2_ to molecular oxygen, thereby generating an electrochemical gradient (Δ pH and ΔΨm) across the inner mitochondrial membrane driving ATP synthesis. Although such transfer of electrons is highly efficient and well coordinated, a small proportion of oxygen is partially reduced to superoxide ion (O_2_^**.**^) in this process [64]. Superoxide production is further augmented by the phosphorylation of electron transport proteins under certain pathophysiological conditions [65, 66]. Superoxide is a highly reactive species and as a remedial measure, mitochondria also contain superoxide dismutase that converts it into H_2_O_2_, its less reactive counterpart [67]. Components of the electron transport chain contribute towards ROS generation under different metabolic and pathophysiological contexts [68]. In post-ischemic heart, generation of superoxide causes decreased S-glutathiony-lation and electron transport activity of Complex II, resulting in mitochondrial dysfunction [69]. Following ischemia-reperfusion, enhanced ROS generation in mitochondria results in thiol modifications in Complex I followed by respiratory dysfunction [70] Right ventricular heart failure induced by pulmonary arterial hypertension is associated with an increased generation of ROS by NADPH oxidase and mitochondrial complex II [71]. In agreement with the deleterious consequences of mitochondrial ROS generation, reduction in oxidative stress by calorie restriction or over expression of catalase augments cardiac performance [72, 73].

### F HIF-1α and Oxygen Sensing

Due to the essentiality of aerobic metabolism and differential oxygenation of tissues, mammals have a highly sensitive mechanism of adapting to altered tissue oxygen level [7, 23, 24]. Thus in addition to the immediate systemic response to hypoxia (as discussed above), all mammalian cells also undergo a long term adaptation by reprogramming its gene expression, hallmark of which is the enhancement of Hypoxia inducible factor-1 or HIF-1 activity [74-76]. Originally, HIF-1 was identified as the activator of erythropoietin gene transcription under hypoxic condition [77, 78]. It is a heterodimeric transcription factor comprising of two polypeptide subunits i.e., α and β. While the α subunits have multiple variants generated from three different genes, the β subunit is ubiquitously expressed [79]. HIF-1 elicits hypoxic response through the hypoxia response element (HRE) located in the regulatory regions of hypoxia responsive genes. Recent data suggests that HIF-1 up- and down regulate several hundred human genes involved in anaerobic glycolysis, tissue oxygenation, vasodialation etc [80]. Although HIF-1 can also be activated by stimuli other than hypoxia; over the years its activation has evolved as a paradigm of hypoxic signaling under various pathophysiological contexts [74-76, 79, 80]. 

### G Mitochondrial Reactive Oxygen Species Generation and Activation of HIF-1α 

Although the role of mitochondrial reactive oxygen species in the activation of HIF-1 was first revealed in late nineties, the precise connection between the two processes had often been debated [81-86]. Nevertheless, three recent studies using tools of (a) RNAi to suppress expression of the constituents of mitochondrial complex III, (b) novel ROS-sensitive FRET probe and (c) murine embryonic cells lacking cytochrome c; have convincingly established the cellular oxygen levels-mitochondrial ROS generation-HIF-1 axis [63, 87-90]. 

Although intracellular generation of ROS/RNS has long been considered to be deleterious for proteins and other macromolecules; their apparent roles in cell signaling had subsequently resulted in a paradigm shift in redox biology [91-94]. It now appears that ROS/RNS might cause momentary but specific modifications of amino acid side chains, thereby imparting molecular switches modulating their functions [95, 96]. Generation of ROS/RNS has accordingly been attributed to the regulation of a number of cell signaling/gene regulatory modules including MAP kinases, PI3-kinase/Akt, transcription factors AP-1, Nrf-2 and NFkB [96-98]. However, due to the transient nature of their effects, only a few direct targets of ROS/RNS have yet been identified [95]. In this regard, studies on the activation of HIF-1α under hypoxia have created an excellent framework of understanding the role of ROS/RNS in hypoxic signaling. Accumulated evidences suggest that under normoxia, a family of prolyl hydroxylases catalyze the hydroxylation of specific proline residues on HIF-1α. Hydroxylated HIF-1α then binds to von Hippel-Lindau (pVHL) tumor suppressor protein that acts as an adapter for the E3 ubiquitin ligase. The ubiquitinylated HIF-1α is then degradaded by the proteasome [99]. These prolyl hydroxylases being dioxygenases requiring oxygen (and 2-oxoglutarate) as substrates and ferrous iron (maintained by ascorbate) as cofactors, provide the missing link between hypoxia, mitochondrial reactive oxygen species generation and stabilization of HIF-1α [100, 101]. It is hypothesized that during hypoxia, the life span of otherwise transient ubisemiquinone radical (a constituent of the electron transport chain) is extended and it transfers its electrons to molecular oxygen dissolved in the mitochondrial membrane, generating superoxide (O_2_^.-^) radicals [63, 102]. Under normal condition O_2_^.-^ radical is attenuated by superoxide dismutase, but when generated in excess, it escapes this attenuation process and further generates other reactive oxygen/nitrogen species [103]. Prolyl hydroxylases are sensitive to ROS generation, though the mechanism is not fully understood as yet. One possibility is that it is due to the oxidative inactivation of the associated iron atom. Therefore, under hypoxic environment, excess generation of superoxide radicals inactivates prolyl hydroxylases, thereby stabilizing  HIF-1. 

### H Myocardial Hypoxia and HIF-1

As in other tissues**, **HIF-1 also plays a major role in eliciting hypoxic response of the mammalian myocardium [104, 105]. Mammalian expression of the regulatory (α) subunit of HIF-1 and it modulator, prolyl hydroxylase significantly vary from one tissue to another while the presence of HIF-1 in the hypoxic myocardium has been confirmed by a number of laboratories [106–110]. Paradoxically, some studies had also documented the abundance of HIF-1α transcripts but not the protein in rat myocardium, implying some additional mechanisms of stabilizing the HIF-1α subunit [105, 111-113]. Nevertheless, in agreement with the proposed role of HIF-1 in mediating hypoxic response in the myocardium, increased expression of certain bona-fide targets genes like that of MCT4 (mediator of lactic acid efflux); BNP (Brain-type natriuretic Peptide, modulator of hemodynamic function and cytoprotection); VEGF (pro-angiogenic factor) and apelin (cardioprotective) have been reported [114-117]. It is thus likely that in the myocardium, HIF-1 expression might be under more stringent regulation involving factors other than oxygen tension alone [118-121]. In agreement, it has recently been reported that acetylcholine can independently but additively (to hypoxia) activate HIF-1 in cardiac myocytes [122]. 

In view of such evident association of cardiac hypoxia and HIF-1 activity, it is imperative that attempts have been made for boosting cardiac function by therapeutic modulation of HIF-1α in the ischemic myocardium. We recently have demonstrated that mice injected with recombinant adenovirus encoding a cardioprotective angiogenic peptide PR39 had protection against ischemia-reperfusion injury that was abolished by the attenuation of HIF-1α by the expression of its dominant negative form [123]. Similarly, transgenic mice having HIF-1α overexpression had diminished infarct size, increased capillary density and enhanced VEGF and iNOS expression following myocardial infarction [124]. Also, activation of HIF-1 by pharmacological or siRNA mediated inhibition of prolyl hydroxylase resulted in significant reduction in myocardial infarct size and expression of chemokines like MIP-2, KC, monocyte chemoattractant protein-1 and ICAM-1, implicating HIF 1 in modulating I/R-associated cardiac inflammatory responses [125].

### I Myocardial Redox Homeostasis and HIF-1

It has long been documented that myocardial ischemia-reperfusion leads to oxidative stress [126-128]. During ischemia, decreased respiration leads to reduced electron transport, causing electron leakage and O_2_^.- ^generation in the mitochondria. Following reperfusion, NO is generated that interacts with O_2_^.-^ ions, producing highly reactive peroxynitrite [103, 129]. A group of enzymes and low molecular weight peptides viz., thioredoxin and glutaredoxin oxidoreductases play a key role in restoring protein thiols under oxidative stress [103, 126-128, 130-132]. Thioredoxin and glutaredoxin are capapable of acting upon a wide range of oxidized proteins, restoring their thiols and thereby maintaining intracellular redox homeostasis, especially in the mitochondrial electron transport system [133, 134]. Since HIF-1 is activated by reactive oxygen species while thioredoxins are involved in maintaining the redox equilibrium, it is likely that the later might play a critical role in modulating HIF-1 in heart in heart (and other tissues). However, our current knowledge about modulation of HIF activities by thioredoxin is quite sporadic. As an example, according to one study, overexpression of thioredoxin-1 in human breast carcinoma MCF-7 cells results in a significant increase in HIF-1α protein (but not RNA) levels under both normoxic and hypoxic conditions [135]. In a more recent study, overexpression of thioredoxin 2 (mitochondria-located) diminished HIF-1α accumulation while that of thioredoxin 1 (cytosolic) enhanced it. Further analysis suggested that thioredoxins affected the translation of HIF-1α while a mitochondria specific antioxidant MitoQ reversed the thioredoxin 2 mediated accumulation of HIF-1α, thereby indicating a complex role of reactive oxygen species in this process [136]. Taken together, the precise regulation of redox equilibrium and HIF-1 activity in heart is an emerging area and it is likely that coming days will shed more light on to it. 

### J Hypoxic Preconditioning and HIF-1

Ischemic preconditioning (IP) is a phenomenon where brief repetitive cycles of ischemia-reperfusion render the myocardium more resistant towards subsequent ischemic insult. Although biochemistry, pharmacology and molecular biology of IP have been extensively investigated, the contribution of HIF-1 in this process is largely unexplored. However, accumulating evidences suggest active involvement of HIF-1 in mediating the preconditioning response [137]. In rat, administration of cobalt chloride, a hypoxic mimetic, activates HIF-1 followed by cardioprotection [138]. In mouse, cardio-protection induced by intermittent hypoxia is attenuated upon targeted inactivation of *HIF-1α* gene, which then can be restored by erythropoietin, a known target of HIF-1 [139]. Further, ischemic preconditioning in rat heart enhances VEGF gene expression and angiogenesis, hallmarks of HIF-1 activity [140]. Upon hypoxic preconditioning, young male rats show upregulation of a battery of genes including *hif-1* and its target *vegf* [141]. Taken together, HIF-1 is likely to be a major contributor towards IP and might be considered for future therapeutic induction of pre-conditioning effect in the myocardium.

### K Conclusions and Future Perspective

**Fig. (1):**
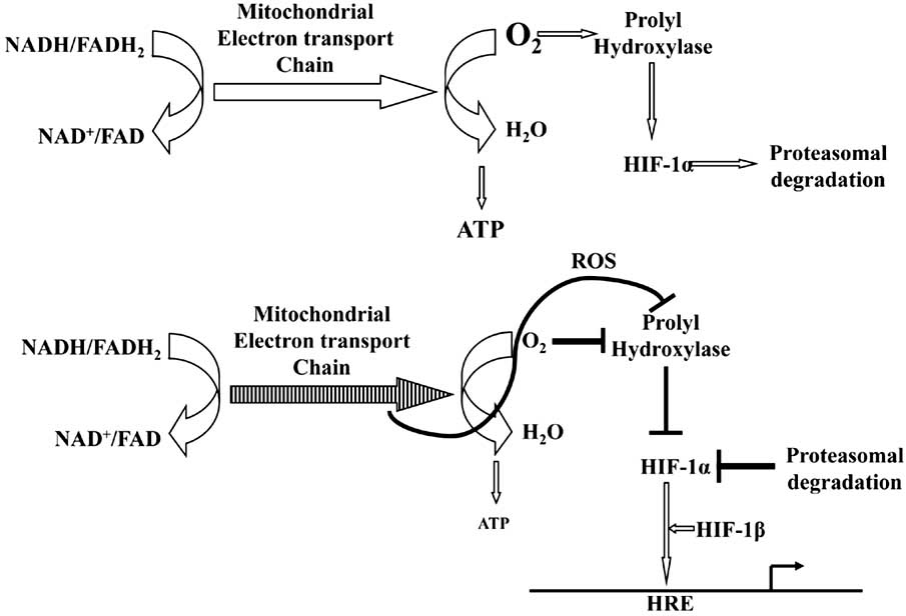
**Schematic representation of hypoxic response by HIF-1: Upper panel**: Under normoxic condition, electron transport chain efficiently reduces oxygen and generates ATP. Also, available oxygen is used by prolyl hydroxylase to hydroxylate HIF-1α. Hydroxylated HIF- 1α then binds to pVHL followed by ubiqutinylation and degradation by proteasome. **Lower panel**: Under hypoxic condition, electron transport is perturbed leading to O_2_^.^ generation. Reduced oxygen supply and to O_2_^.^ inhibit prolyl hydroxylase activity leading to the stabilization of HIF-1α. HIF-1α then dimerize with HIF-1β, binding to its target HRE and activating a battery of hypoxia responsive genes with diverse physiological consequences as shown.

Our interest in cellular and organismal oxygen sensing attained further height when the criticality of oxygen levels in the etiology diseases like cancer, cardiac ischemia and stroke became apparent. Although our present knowledge about hypoxic response were largely derived form the framework of tumor angiogenesis, parallel progress has also been made in the context of ischemic and infracted myocardium (Fig. **1**). However, whether hypoxic responses are tissue differential or not and if so, to what extent, is not clear as yet [142]. In this context, certain unexpected observations deserve special attention. In a recent study, adult mice with late-stage brain specific deletion of HIF-1α showed protection from acute hypoxia induced cell death, suggesting more divergent role(s) of HIF-1 (or redundant roles of HIF-2/3) than originally anticipated [143]. It is thus expected that similar analysis in the myocardium might further enhance our knowledge about the mechanism of hypoxic response in a tissue specific context. Another area of potential importance (and future debate) is the precise role of ROS in mediating oxygen sensing and eliciting the hypoxic response. Although a critical role of mitochondrial ROS in modulating prolyl hydroxylase activity and stability of HIF-1 is now generally accepted, the precise mechanism of ROS generation and its difference from those generated under other oxygen associated setups like hyperoxia, preconditioning etc. is yet to be deciphered [7, 63, 144]. Finally, further study on the additional mechanisms of hypoxic response such as translational control and post-translational modification of signaling kinases will contribute towards a comprehensive understanding and therapeutic intervention of ischemic heart diseases [66, 145]. 

## ABBREVIATIONS

ATP: = Adenosine mono phosphate
AMP Kinase: = AMP-activated protein kinase
AP-1: = Activator protein-1
bHLH: = Basic-helix-loop-helix
BNP: = Brain natriuretic peptide
FRET: = Fluorescence resonance energy transfer
HIF-1: = Hypoxia inducible factor 1
I/R: = Ischemia-reperfusion
ICAM-1: = Intercellular cell adhesion molecule
IGF: = Insulin like growth factor
IL-6: = Interleukin 6
iNOS: = Inducible nitric oxide synthase
IP: = Ischemic preconditioning
MAP Kinase: = Mitogen activated protein kinase
MCT4: = Monocarboxylate transporter 4
MIP-2: = Macrophage Inflammatory Protein-2
Mn-SOD: = Superoxide dismutase
NADPH: = Nicotinamide adenine dinucleotide phosphate
NCX: = Neural crest homeobox
NFkB: = Nuclear factor-kappa B
NO: = Nitric oxide
PAS: = Per/Arnt/Sim
PI3 Kinase: = Phosphoinositide 3-kinase
RNS: = Reactive nitrogen species
ROS: = Reactive oxygen species
RyR: = Ryanodine receptors
VEGF: = Vascular endothelial growth factor
